# IUC24413-79 Comparative performance of AI vs radiologists in pre-biopsy mpMRI prostate cancer diagnosis: a systematic review and meta-analysis of multi-centre, multi-vendor studies

**DOI:** 10.1093/oncolo/oyaf248.002

**Published:** 2025-08-29

**Authors:** Uzair Khan, Aaditya Tiwari, Karran Bhagat, Tulika Nahar, Soumya Arun, Aruni Ghose, Abel Tesfai, Alexandra Naranjo, Pinky Kotecha, Nicolas Omorphos, Joecelyn Kirani Tan, Maryam Hasanova, Akash Maniam, Giuseppe Luigi Banna, Yüksel Ürün, Swarupa Mitra, Karan Jatwani, Stergios Boussios, Prasanna Sooriakumaran, Benjamin Lamb, Jeremy Teoh, Antony Rix, Amy Rylance, Balraj Dhesi, Atif Khan, Sola Adeleke

**Affiliations:** Early Cancer Institute, University of Cambridge, Cambridge, United Kingdom; Princess Alexandra Hospital NHS Trust, Harlow, United Kingdom; Royal Surrey NHS Foundation Trust, Guildford, United Kingdom; Queen’s University, Belfast, United Kingdom; Queen Mary University of London, London, United Kingdom; Barts Cancer Centre, London, United Kingdom; Prostate Cancer UK, London, United Kingdom; Prostate Cancer UK, London, United Kingdom; Queen Mary University of London, London, United Kingdom; University Hospitals of North Midlands NHS Trust, Stoke-on-Trent, United Kingdom; University of Manchester, Manchester, United Kingdom; OncoFlowTM, London, United Kingdom; Caribbean Cancer Research Institute, Trinidad and Tobago; Portsmouth Hospitals University NHS Trust, Portsmouth, United Kingdom; Ankara University Cancer Research Institute, Ankara, Turkey; Fortis Cancer Institute, Gurugram, India; George Washington Cancer Centre, Washington, DC, United States; University of Ioannina, Ioannina, Greece; University of Oxford, Oxford, United Kingdom; North East London Cancer Alliance, London, United Kingdom; Chinese University of Hong Kong Medical Centre, Hong Kong; Lucida Medical Ltd, Cambridge, United Kingdom; Prostate Cancer UK, London, United Kingdom; University Hospitals Birmingham NHS Foundation Trust, Birmingham, United Kingdom; Leeds Teaching Hospitals NHS Trust, Leeds, United Kingdom; King’s College London, London, United Kingdom

## Abstract

**Background:**

Multiparametric MRI (mpMRI) is the cornerstone for diagnosing clinically significant prostate cancer (csPC), that is, International Society of Urological Pathology (ISUP) Grade ≥2. The European Association of Urology (EAU) recommends its upfront implementation in biopsy-naive patients to guide prostate biopsy decision making (PMID 38614820). However, significant inter- and intra-observer reporting variability affects patient outcomes. Our objective was to systematically review and evaluate the comparative performance of AI vs radiologists in diagnosing prostate cancer from pre-biopsy prostate mpMRI.

**Methods:**

Our systematic review [PROSPERO ID: CRD420251037432] included MEDLINE, PMC, EMBASE, SCOPUS, and COCHRANE databases. Searching primary research published in 2010 and beyond yielded 6,389 records. After screening 3,747 articles, 137 underwent full-text review, with 3 meeting inclusion criteria—2 from database searches and 1 from grey literature. A meta-analysis using R assessed the diagnostic performance of the AI models, with area under the curve (AUC) as the primary outcome.

**Results:**

Three multi-center, multi-vendor intervention studies on prostate mpMRI compared AI characterization of csPC vs standard of care (SOC), that is, ≥2 radiologists performing Prostate Imaging-Reporting and Data Systems (PI-RADS) version ≥2 scoring. A meta-analysis, including 552 pre-biopsy patients, yielded pooled sensitivity 0.884 (95% CI, 0.75-0.98), specificity 0.681 (95% CI, 0.51-0.80), and AUC 0.837 (95% CI, 0.690-0.950) for the AI—principally supervised machine learning (ML) models.

Model 1 (PMID 40016318) had a 95% sensitivity, 67% specificity and was non-inferior to SOC (AUC 0.91 vs 0.95; *P* = .044). Model 2 (PMID 37345961) reported 86%-91% sensitivity, 64%-75% specificity and was also non-inferior to SOC (comparable AUCs 0.82-0.86). Model 3 (PMID 33671533) showed 89% sensitivity and superiority over SOC (AUC 0.75 vs 0.47). Models 1 and 2 exhibited strong generalizability, with Model 2 aligning closely with PI-RADSv2 lesion characterization.

**Conclusions:**

The mpMRI-directed prostate biopsy pathway increases csPC detection and decreases PC negative biopsy rates. Adopting this implies a significant time and labor-intensive radiology workforce pressure. Our meta-analysis demonstrates how AI PC diagnostic accuracy is comparable to radiologists. This aids standardization, reduces diagnostic variability and radiologist workload.

